# Identification of a Functional Risk Variant for Pemphigus Vulgaris in the *ST18* Gene

**DOI:** 10.1371/journal.pgen.1006008

**Published:** 2016-05-05

**Authors:** Dan Vodo, Ofer Sarig, Shamir Geller, Edna Ben-Asher, Tsviya Olender, Ron Bochner, Ilan Goldberg, Judith Nosgorodsky, Anna Alkelai, Pavel Tatarskyy, Alon Peled, Sharon Baum, Aviv Barzilai, Saleh M. Ibrahim, Detlef Zillikens, Doron Lancet, Eli Sprecher

**Affiliations:** 1 Department of Dermatology, Tel-Aviv Sourasky Medical Center, Tel-Aviv, Israel; 2 Department of Human Molecular Genetics and Biochemistry, Sackler Faculty of Medicine, Tel-Aviv University, Tel-Aviv, Israel; 3 Department of Molecular Genetics, Weizmann Institute of Science, Rehovot, Israel; 4 Department of Dermatology, Sheba Medical Center, Tel-Hashomer, Israel; 5 Institute of Experimental Dermatology, University of Luebeck, Luebeck, Germany; 6 Department of Dermatology, University of Luebeck, Luebeck, Germany; University of Pennsylvania, UNITED STATES

## Abstract

Pemphigus vulgaris (PV) is a life-threatening autoimmune mucocutaneous blistering disease caused by disruption of intercellular adhesion due to auto-antibodies directed against epithelial components. Treatment is limited to immunosuppressive agents, which are associated with serious adverse effects. The propensity to develop the disease is in part genetically determined. We therefore reasoned that the delineation of PV genetic basis may point to novel therapeutic strategies. Using a genome-wide association approach, we recently found that genetic variants in the vicinity of the S*T18* gene confer a significant risk for the disease. Here, using targeted deep sequencing, we identified a PV-associated variant residing within the *ST18* promoter region (p<0.0002; odds ratio = 2.03). This variant was found to drive increased gene transcription in a p53/p63-dependent manner, which may explain the fact that ST18 is up-regulated in the skin of PV patients. We then discovered that when overexpressed, ST18 stimulates PV serum-induced secretion of key inflammatory molecules and contributes to PV serum-induced disruption of keratinocyte cell-cell adhesion, two processes previously implicated in the pathogenesis of PV. Thus, the present findings indicate that ST18 may play a direct role in PV and consequently represents a potential target for the treatment of this disease.

## Author Summary

Pemphigus vulgaris is a life-threatening autoimmune skin blistering disease. A large body of evidence indicates that the propensity to develop this condition is in part genetically determined. Using a genome wide association approach, we recently identified pemphigus vulgaris-associated genetic variations in the vicinity of the *ST18* gene. In the present study, we identify a risk variant residing within the *ST18* promoter region which drives *ST18* gene promoter activity in a p53/p63-dependent manner, which is in line with the fact that ST18 is up-regulated in the skin of PV patients. Using functional assays, we show that ST18 overexpression increases PV serum-induced expression of pro-inflammatory mediators, as well as augments PV serum-induced disruption of keratinocyte cell-cell adhesion, which are hallmarks of pemphigus pathogenesis. Our findings therefore support a direct role for ST18 in the pathogenesis of pemphigus vulgaris, and position ST18 as a new molecular target of potential interest for the treatment of disease. From a broader perspective, these observations underscore the importance of genetic variations affecting the susceptibility of target tissues to autoimmunity.

## Introduction

Pemphigus refers to a group of autoimmune blistering disorders which affect mucocutaneous tissues [1,2]. Pemphigus vulgaris, the most common subtype of the disease, is estimated to have a worldwide annual incidence of 0.76–6.7 new cases per million [1] and is between 4- to 10-fold more common among Jews as compared with other populations [3]. The disease is characterized by the development of flaccid blisters over the skin and mucosal surfaces, which rupture easily to form large painful erosions with little tendency to heal and which, if left untreated, increase the probability of life-threatening complications [1]. Since the advent of corticosteroid treatment, mortality has dropped to 10%, though morbidity is still considerable [1].

PV is traditionally considered to result from abnormal desmosome function caused by circulating auto-antibodies (auto-Abs) directed against desmosomal antigens, mainly desmoglein (Dsg) 3 and Dsg1 [2], which in turn leads to loss of adhesion (acantholysis) between keratinocytes. More recently, additional pathogenetic mechanisms, not directly involving desmosome destabilization, have also been suggested to be operative in PV IgG-induced blister formation, such as activation of apoptosis; increased pro-inflammatory cytokine secretion; aberrant cell-cell signaling; and activation of muscarinic receptors uniquely expressed by basal keratinocytes [4]. The propensity to develop the disease is believed to be to a large extent genetically determined as attested by familial occurrence of PV, the presence of circulating PV IgG Abs in healthy first-degree relatives of PV patients and ethnic clustering [3,5–7]. This in turn offers the possibility to identify elements of importance to PV etiology through a genetic approach. We recently conducted a genome wide association study (GWAS) in a genetically homogenous population of Jewish extraction, followed by replication in two cohorts, and identified an association between PV and the *ST18* gene locus [8]. Although *ST18* encodes a transcription factor possibly regulating apoptosis and inflammation [9], two processes of potential relevance to PV [10,11], it is not clear whether this genetic association reflects causal involvement of *ST18* in PV pathogenesis. In the present study, we examined the possibility that *ST18* plays a direct role in PV pathogenesis.

## Results

### Identification of a PV-associated functional variant in the ST18 gene locus

**Fig 1:**
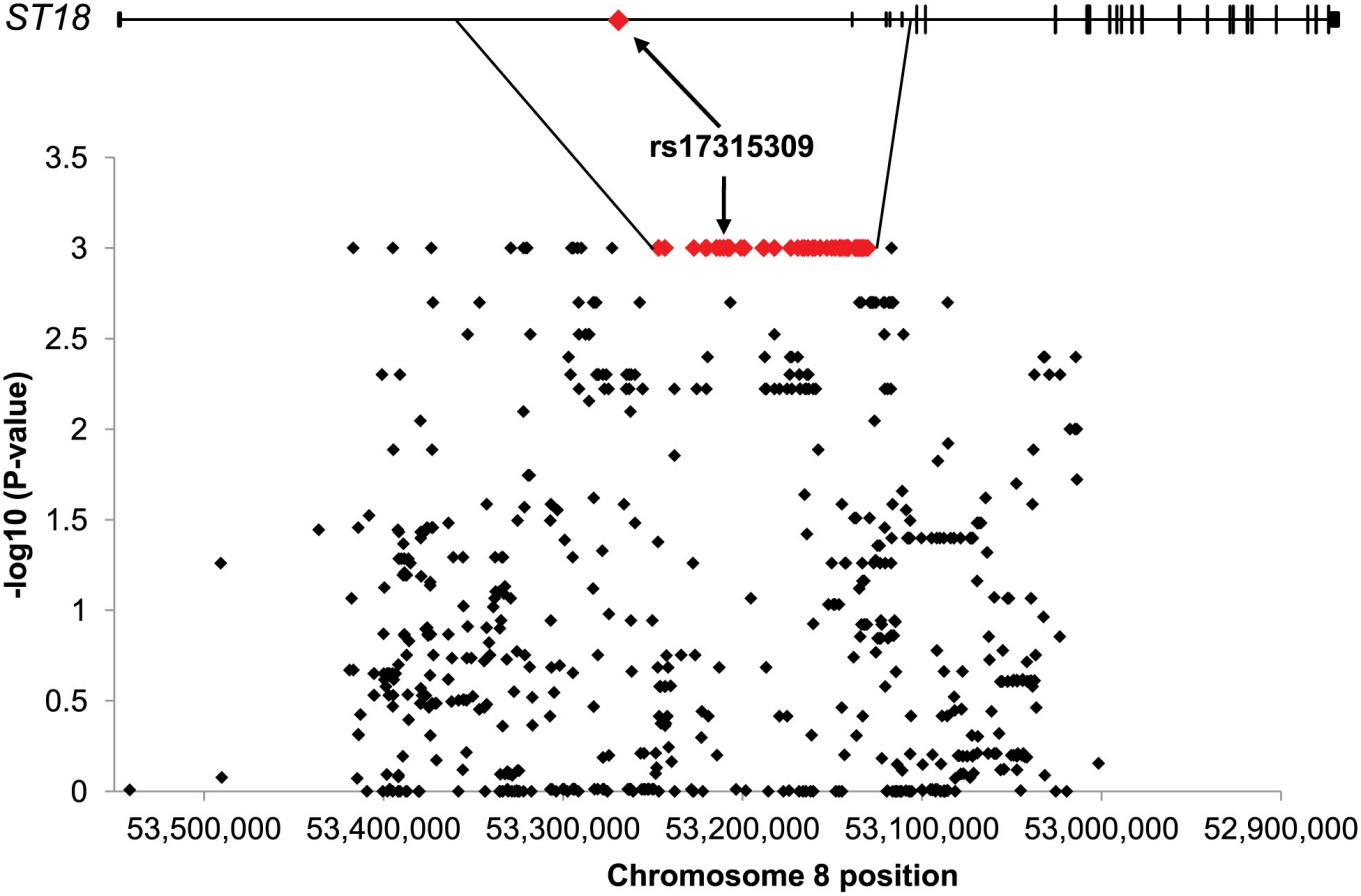
Targeted deep sequencing of the ST18 gene locus. Deep sequencing of the ST18 gene locus in 16 PV patients led to identification of 789 genetic variants (black dots), depicted along the ST18 gene locus (X axis, chr8:53,023,392–53,373,519, GRCh37/hg19 assembly; Y axis, negative log-transformed P-values of association score). A case-control association analysis against the 1000 genome project data revealed a large PV-associated haplotype block (in red) residing within an ST18 intron and harboring rs17315309 (arrows).

Given the fact that a previous GWAS demonstrated an association between PV and variants in the vicinity of the *ST18* gene locus [8], we aimed at characterizing this risk region and therefore performed targeted deep sequencing of the *ST18* locus in 16 Jewish PV patients, initially comparing the sequencing results to the 1000 Genomes Project (1000GP) data (http://www.1000genomes.org). Since PV is a complex disease, our primary targets were common single nucleotide polymorphisms (SNPs) that were enriched in the sequenced cohort as compared with controls. We therefore examined all SNPs with non-zero frequencies in the public databases that were not in repetitive regions, totaling 789 SNPs (Fig 1). A case-control association analysis for each SNP, using chi-square and permutation test, led to the identification of a genomic haplotype block strongly associated with the propensity to develop PV (p<0.001) (Fig 1 and S1 Fig). We discovered that this risk haplotype harbors two variants previously found to be most significantly associated with PV in the original GWAS, rs4074067 and rs2304365 [8]. Collectively, the SNPs found within the risk haplotype block had a frequency of 50% in the sequenced cohort and of only 8% in the total 1000GP population.

Within the risk haplotype block, we identified a genetic variant, rs17315309 (Fig 1), which displayed significant association to PV based on the deep sequencing data. We then replicated this association in an independent set of 185 Jewish PV patients compared with 183 population-matched healthy controls (p<0.001; S1 Table).

**Fig 2:**
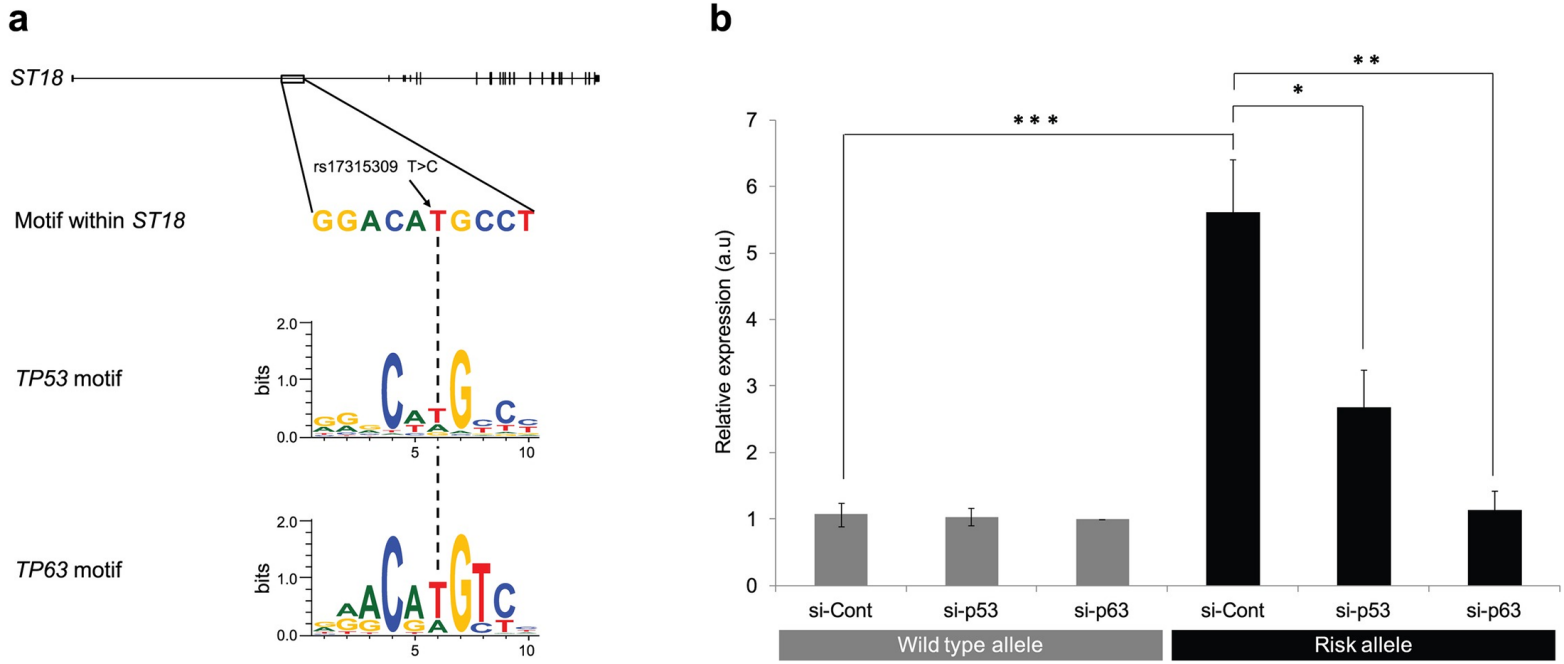
Characterization of a PV-associated functional allele. (a) rs17315309 resides within a p53/p63 half binding site, upstream to the ST18 coding sequence, and results in the substitution of a highly conserved T nucleotide (broken arrow) within the consensus binding sites of p53 and p63 (MotifMap database; http://motifmap.ics.uci.edu/); (b) NHEKs were co-transfected with a luciferase reporter construct under the regulation of a 282 bp fragment from the putative ST18 promoter, harboring either the wild type or the risk allele of rs17315309, and with control (si-Cont), TP53- (si-p53) or TP63- (si-p63) specific siRNAs. Luciferase activity measurements are provided as arbitrary units (a.u.) relative to the luciferase activity measured in cells transfected with wild type allele and control siRNA. Results represent the mean of three independent experiments ± SE (*p<0.05, **p<0.01 and ***p<0.001 by 2-tailed t test).

A number of bioinformatic analyses suggested that this variant may be of functional importance and possibly up-regulate *ST18* promoter activity. First, using HMR Conserved Transcription Factor Binding Site database, implemented in the UCSC Genome Browser (https://genome.ucsc.edu/), this variant was found to reside within a p53 transcription factor binding site consensus sequence (Fig 2a). The p53 binding motif consists of two very similar, closely located, half-sites, each 10 bp long [12,13], although it has been shown that one decamer is sufficient for p53 or p63 binding and activity [14–19]. Second, the binding motif harboring rs17315309 is located in an intron of *ST18*, upstream to the gene coding sequence and inside a 170 bp long DNAse hypersensitivity cluster (chr8:53207581–53207750, ENCODE; http://genome.ucsc.edu/ENCODE/), lending further support to the possibility that this region plays a regulatory role. Of note, p53 is known to recognize consensus binding motifs located proximal to the transcription start site of target genes, either within the promoter region or a gene intron [20]. Third, using the 100 Vertebrae Conservation by PhastCons, implemented in the UCSC Genome Browser (http://compgen.cshl.edu/phast/), we found that both rs17315309 and the p53/p63 binding motif are highly conserved with a maximum conservation score of 1 (range 0 to 1). Similarly, using Biobase Transfac Matrix Database (v7.0), implemented in the UCSC Genome Browser (http://www.gene-regulation.com/pub/databases.html), the 10 bp long binding motif containing rs17315309 was found to be remarkably conserved (computed score: 992; maximal score: 1000), supporting the possibility that it represents a biologically functional binding site. Lastly, as rs17315309 results in a T to C substitution at position 6 of the p53/p63 binding motif, we wished to examine the conservation of this nucleotide in the binding site consensus sequence. Both MotifMap (http://motifmap.ics.uci.edu) and Jasper (http://jaspar.genereg.net) databases indicated that position 6 in the half-binding site consensus sequence of both p53 and p63 is highly conserved and consists usually of a T nucleotide with a minimum to no abundance of a C nucleotide (Fig 2a). Taken collectively, these data suggested that modification of the wild type rs17315309 allele within the p53/p63 binding site may be of biological significance.

To examine this possibility, we transfected normal human keratinocytes (NHEKs) with a PGL4.17 luciferase reporter construct under the regulation of a 282 bp fragment spanning the p53/p63 binding motif containing either the wild type (T) or the risk (C) rs17315309 allele. The C allele was found to induce a more than 5-fold increase in luciferase activity (*p*<0.001) (Fig 2b). Moreover, when the construct was co-transfected with p53- or p63-specific siRNAs (S2a and S2b Fig), this effect was markedly attenuated (Fig 2b), indicating that the PV-associated rs17315309 risk allele increases *ST18* promoter activity in a p53-/p63-dependent manner. These results are in line with previous data showing that a single nucleotide change in a canonical p53/p63 binding sequence is enough to affect p53 or p63 binding [21].

### ST18 promotes PV IgG-induced keratinocyte secretion of pro-inflammatory mediators

Given these data, the physiological roles of ST18 and the fact that ST18 is markedly overexpressed in the non-lesional epidermis of PV patients [8], we sought to ascertain the consequences of ST18 overexpression on pathophysiological hallmarks of the disease.

**Fig 3:**
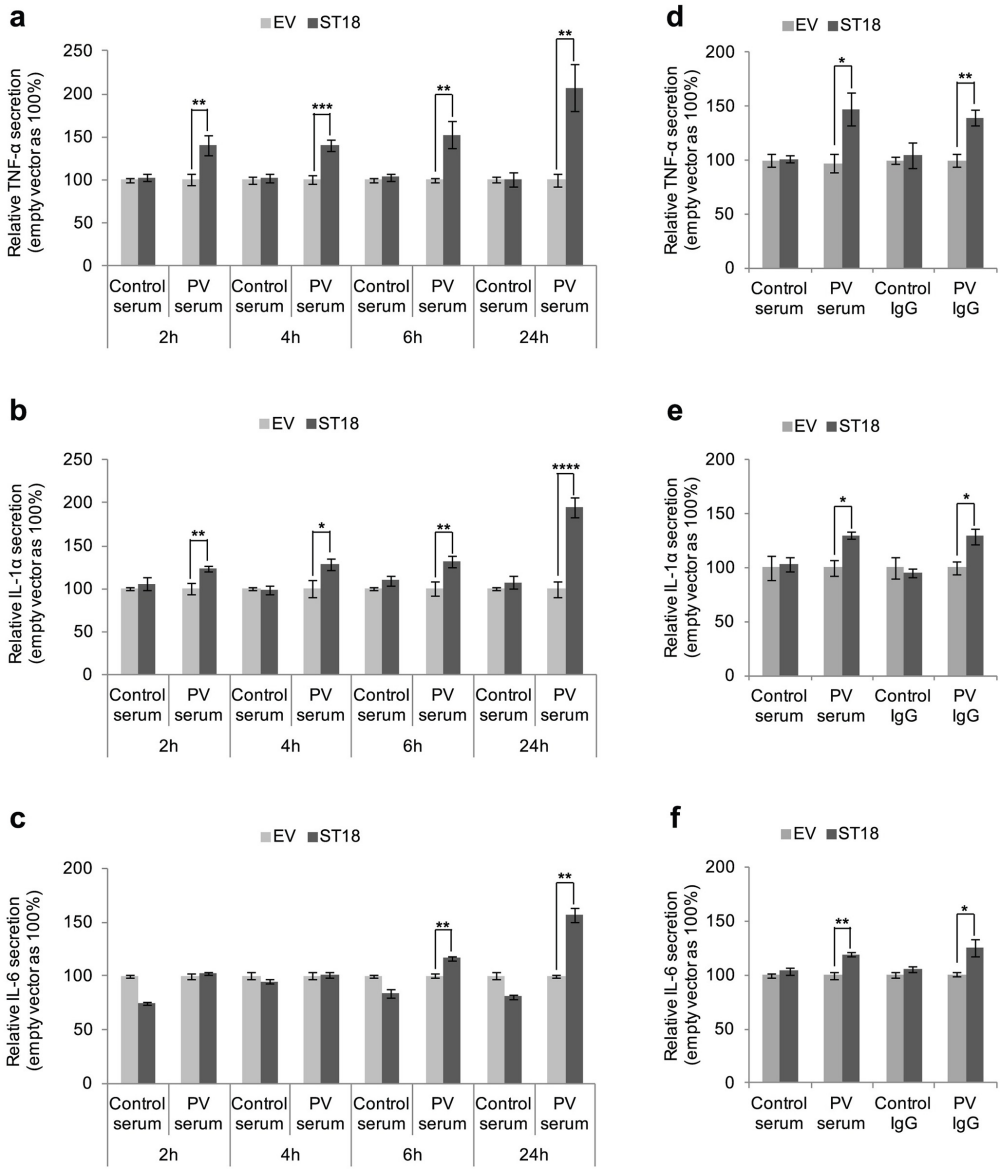
ST18 promotes PV serum- and PV IgG-induced release of pro-inflammatory cytokines. NHEKs were transfected with an ST18 expression vector (ST18, dark grey) or control empty vector (EV, light grey). (a,b,c) Supernatants were collected 2, 4, 6 and 24 hours post exposure to PV serum or control serum and (a) TNFα, (b) IL-1α and (c) IL-6 secretion was measured as described in Materials and Methods; (d,e,f) Supernatants were collected 6 hours (IL-1α and IL-6) or 24 hours (TNFα) post exposure to PV and control serum or PV and control IgG and (d) TNFα, (e) IL-1α and (f) IL-6 secretion was measured as described in Materials and Methods. Results represent the mean of three independent experiments and are expressed as the relative cytokine secretion in percentage, compared to control (empty vector) ± SE (*p<0.05, **p<0.01, ***p<0.001 and ****p<0.0001 by 2-tailed t test).

We first examined the effect of ST18 on keratinocyte secretion of pro-inflammatory cytokines which are believed to contribute to PV disease phenotype [1,4,11]. Overexpression of ST18 (S2c Fig) in the presence of normal serum or control IgG did not affect the secretion of either TNFα, IL-1α or IL-6 (Fig 3). In contrast, when overexpressed in the presence of PV serum, ST18 was found to drive the secretion of all three cytokines (Fig 3a–3c), indicating that ST18 functions by promoting PV-induced keratinocyte secretion of pro-inflammatory cytokines. This effect was seen early with TNFα and IL-1α but late with IL-6 (Fig 3a–3c). We then repeated the same experiments, comparing the effect of control and PV sera to the effect of control IgG and PV IgG. Overexpression of *ST18* was found to increase PV serum-induced and PV IgG-induced secretion of TNFα, IL-1α and IL-6, to the same extent, while not affecting the secretion of these cytokines in the presence of control serum or control IgG (Fig 3d–3f).

### ST18 augments PV IgG-induced keratinocyte cell-cell disadhesion

**Fig 4:**
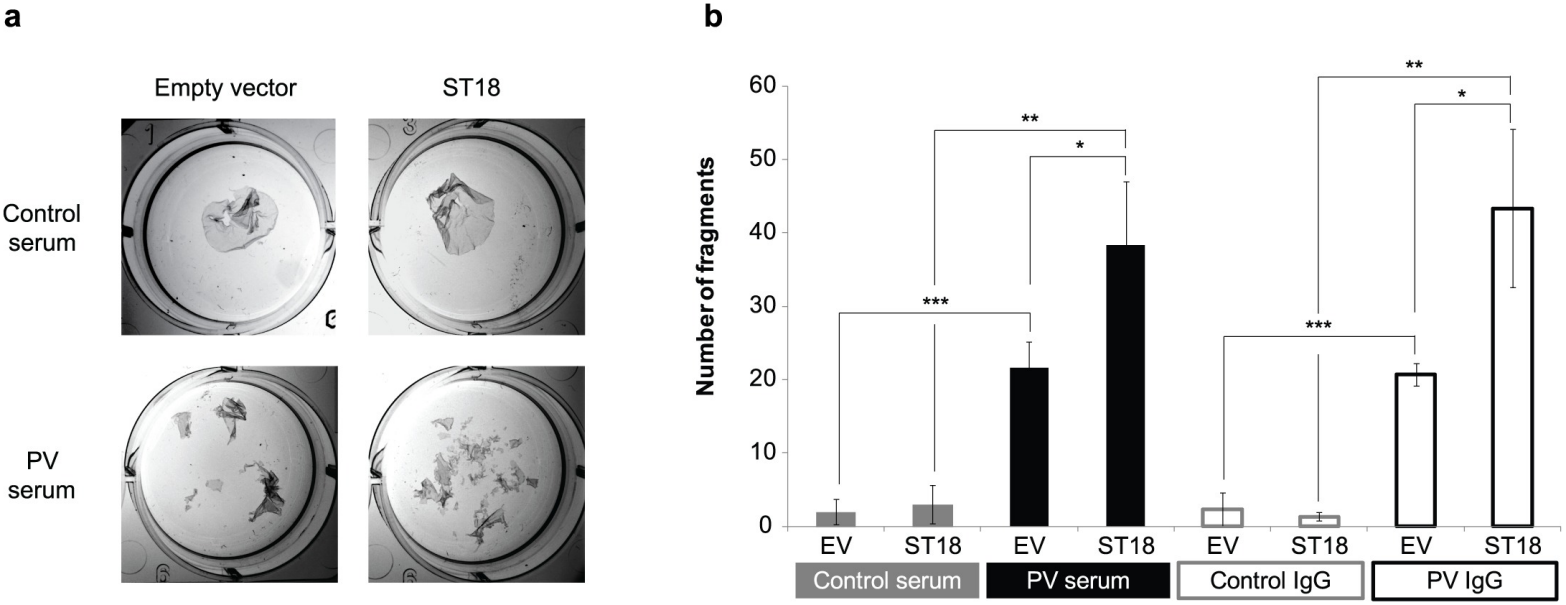
ST18 promotes PV serum and PV IgG-induced cell-cell disadhesion. We used a dispase-based dissociation assay to evaluate the effect of ST18 overexpression on cell adhesion. NHEKs transfected with a ST18 expression vector (ST18) or with a control vector (EV) were grown to confluency in the presence of PV serum and control serum or PV IgG and control IgG. (a) Epidermal sheets were released from the tissue plates and subjected to mechanical stress as described in Materials and Methods; (b) the resulting fragments were counted. Results represent the mean of three independent experiments and are expressed as number of fragments ± SE (*p<0.05, **p<0.01, ***p<0.001 by 2-tailed t test).

As disruption of epidermal cell-cell adhesion is a pathogenic hallmark of PV [1], we investigated ST18 effect on PV serum-induced cell-cell disadhesion. For this purpose, we used the dispase-based dissociation assay. In this system, PV serum destabilizes intercellular bonds, compromising epidermal sheet resilience to mechanical stress [22] (Fig 4a). NHEKs overexpressing ST18 and exposed to PV serum exhibited a more than 2-fold decrease in cell-cell adhesion, as compared to cells exposed to PV serum and transfected with an empty vector (*p<*0.05) (Fig 4b) or with a vector overexpressing a non-relevant gene (S3 Fig). The deleterious effect of ST18 overexpression on cell-cell adhesion was similar when cells were exposed to pooled PV sera or to PV IgG (Fig 4b). Taken together, these results demonstrate that ST18 may also contribute to PV pathogenesis by potentiating PV IgG-induced acantholysis.

## Discussion

Despite a large body of epidemiological evidence supporting a role for genetic elements in determining the propensity to develop the disease [3,5–7], little is currently known about the genetic basis of PV. Using a GWAS approach, we previously identified an association between PV and a genomic segment on chromosome 8q11 spanning the *ST18* gene [8]. *ST18* encodes the suppression of tumorigenicity 18 (ST18), a 115kD member of the myelin transcription factor 1 (MyT1) family of transcription factors containing several zinc-finger DNA-binding domains [23]. ST18 is constitutively expressed in the brain, and less so in the heart, liver, kidney, skeletal muscle, pancreas, testis, ovary and prostate [24]. ST18 is undetectable in normal skin, but is significantly expressed in the epidermis of PV patients [8]. Two recent studies [9,25] demonstrated the role of ST18 in apoptosis and inflammation, two processes of direct relevance to the pathogenesis of PV [10,11]. ST18 was shown to mediate TNFα-induced transcription of pro-apoptotic and pro-inflammatory genes in fibroblasts, including *TNFα*, *IL-1α* and *IL-6* [9].

In the present study, using targeted deep sequencing of the *ST18* locus, we identified within the *ST18* promoter region a PV-associated genetic variant, rs17315309, which was shown to drive gene transcription in a p53/p63-dependent fashion. Of interest PV serum was previously found to induce p53 expression [26] and p63 is overexpressed in the skin of pemphigus foliaceus patients [27].

Together with the fact that ST18 expression is up-regulated in the skin of PV patients [8], these data suggested that ST18 overexpression may be directly contributing to the disease pathogenesis. And indeed, ST18 up-regulation was found to induce the secretion of TNFα, IL-1α and IL-6 in the presence of PV serum as well as in the presence of PV IgG antibodies. The level of all three cytokines has been previously reported to be increased in the serum as well as in the lesional skin and blister fluid of PV patients [11,28–34]. In addition, serum levels of TNFα and IL-6 were shown to negatively influence PV outcome [28,35]. Finally, both TNFα and IL-1α have been reported to be up-regulated by PV IgG and to contribute to PV IgG-induced acantholysis and apoptosis in keratinocytes [32,36].

Most importantly, ST18 was found to potentiate PV IgG-induced cell-cell disadhesion. The mechanism of action of ST18 in inducing cell-cell disadhesion remains to be fully elucidated but may involve some of the cytokines whose secretion was found to be increased in the presence of ST18 overexpression [32,36]. Clearly, other pro-inflammatory factors may also contribute to keratinocyte dissociation. The fact that PV IgG cause secretion of cytokines and loss of cell adhesion to a comparable extent as PV serum demonstrates that ST18 promotes the effect of autoantibodies rather than serum factors on the secretion of cytokines and on loss of cell adhesion.

Taken collectively, our data indicate that a PV-associated risk allele at the *ST18* gene locus may drive ST18 up-regulation which in turn could contribute to PV pathogenesis by stimulating keratinocyte-derived cytokine release and by compromising epidermal cell-cell adhesion. Thus, ST18 is likely to contribute to PV pathogenesis by increasing keratinocytes susceptibility to the deleterious effects of PV-associated autoantibodies rather than by affecting the production of these antibodies. Supporting this possibility, we did not detect any effect of ST18 genotype on anti-Dsg3 ELISA status in a series of PV patients (S4 Fig). The present results therefore underscore the importance of genetic variations affecting target tissues in the pathogenesis of inflammatory diseases as previously shown for other skin disorders [37,38].

## Materials and Methods

### Ethics statement

The study was conducted according to a protocol approved by our institutional review board and the National Committee for Genetic Studies of the Israeli Ministry of Health in accordance with the Declaration of Helsinki Principles (102-2006/TLV-0537-15). All family members provided written informed consent to participate in this study

### Patients

All family members provided written informed consent to participate in this study. Blood samples were obtained from all participants according to a protocol approved by our institutional review board and the National Committee for Genetic Studies of the Israeli Ministry of Health in accordance with the Declaration of Helsinki Principles. The diagnosis of PV was posed based upon clinical features, suprabasal separation on histology, positive direct and indirect immunofluorescence microscopy, and/or ELISA detection of anti-Dsg Abs. Genomic DNA was extracted from peripheral blood leukocytes using the 5 Prime ArchivePure DNA Blood kit (5 Prime Inc., Gaithersburg, MD, USA).

### PV sera and PV IgG preparation

We used two different mixes of pooled sera from newly diagnosed PV patients (n = 3 and 4) with active disease and an anti-Dsg3 titer above122 relative units/ml, as measured by the anti-desmoglein 3 ELISA (IgG) test kit (Euroimmune AG, Luebeck, Germany). The sera were obtained prior to the initiation of any systemic immunosupressive treatment. PV IgGs were purified as previously described [39] and used at a final concentration of 65 μg/ml.

### Targeted deep sequencing of the ST18 locus

DNA enrichment was performed using HaloPlex kit (Agilent Technologies, Santa Clara, CA, USA) and sequencing was conducted on a MiSeq system sequencer (Illumina, San Diego, CA, USA) with 150 bp paired-end reads. A total of 463407 bp were included in the capture design, covering the entire *ST18* gene (chr8: 53,023,399–53,373,519, GRCh37/hg19 assembly) as well as 10 kb downstream and 50 kb upstream to the gene and an additional 2 Mb located upstream and downstream to the gene and predicted to harbor putative regulatory regions (https://www.encodeproject.org).

The sequencing data were processed using MiSeq Reporter 2.0.26 and Casava softwares (Illumina, San Diego, CA, USA) and analyzed for quality control using FastQC software (http://www.bioinformatics.babraham.ac.uk/projects/fastqc). Reads were aligned to the Genome Reference Consortium Human Build 37 (GRCh37/hg19) using Burrows-Wheeler Aligner [40] and variant detection was achieved using The Genome Analysis Toolkit [41]. Variants were annotated by ANNOVAR [42] and the frequency of each variant was determined using data from dbSNP138, the 1000 Genome Project and an in-house database. Case-control association test for variants was performed with chi-square, and permutation test, using the Caucasian population from the 1000 Genome Project data (http://www.1000genomes.org) as a control.

### rs17315309 genotyping

To screen for the rs17315309 allele, we PCR-amplified a 317 bp fragment, with ReddyMix PCR Master Mix (Thermo scientific, NH, USA) and the following primers 5`- TGCTTGCCGTTTGTAAGATG-3`and 5`-AGCCTGGTTCAAGAGCCTTC-3`. Cycling conditions were as follows: 94°C, 4min; 94°C, 30 sec; 61°C, 30 sec; 72°C 30 sec, for 2 cycles, 94°C, 30 sec; 59°C, 30 sec; 72°C 30 sec, for 2 cycles, 94°C, 30 sec; 57°C, 30 sec; 72°C 30 sec, for 38 cycles, 72°C for 10 min. The T allele is associated with the presence of a recognition site for endonuclease NspI (New England Biolabs, Hitchin, UK). After incubation at 37°C for 16 hours followed by 20 min of inactivation at 65°C, the digested PCR products were electrophoresed in ethidium bromide-stained 3% agarose gels.

### Cells cultures

NHEKs were extracted from skin discarded during plastic surgery procedures, after written informed consent had been obtained as previously described [43]. The keratinocytes were seeded on feeder plates containing 3T3-J2 fibroblasts and were grown in medium Green containing 42.5% DMEM (Biological Industries, Beit-Haemek, Israel), 42.5% DMEM/F12 (Biological Industries, Beit-Haemek, Israel), 10% FCII serum, 1% penicillin-streptomycin, 1mM L-glutamine, 1mM sodium pyruvate, 5 μg/mL Insulin, 0.2 mM adenine, 0.5 μg/mL hydrocortisone, 2nM Triiodothyronine, 0.1 nM cholera toxin and 10 ng/mL EGF, and were frozen upon 90% confluence. Cell were then thawed and cultured in KGM media (Lonza, Basel, Switzerland).

For the dispase-based dissociation assay, NHEKs were extracted from foreskin using the same conditions and were thawed and cultured in M154 media (Life Technologies, Carlsbad, CA).

### ST18 overexpression

A 8.0 kb-clone containing the *ST18* open reading frame in a pCMV6-Entry vector (4.9kb) was purchased from Origene Technologies Company (Rockville, MD, USA). The empty pCMV6-Entry was used as a control. Additionally, a 6.6 kb-clone containing the *CNBD2* open reading frame in a pCMV6-Entry vector (4.9kb) was purchased from Origene Technologies Company (Rockville, MD, USA) and used a negative control for protein overexpression. NHEKs were cultured to 80% confluence and subjected to a transient transfection using lipofectamine2000 (Life Technologies, Carlsbad, CA).

### p53/p63 binding site luciferase reporter assay

A 282 bp *ST18* gene fragment spanning rs17315309 was PCR-amplified using ReddyMix PCR Master Mix (Thermo scientific, NH, USA), primers 5’-AAAATTAGGTACCGCGTTCAAGCACTCTATTACCT-3’ and 5’-AAAAGGACTCGAGGCTTGCCGTTTGTAAGATGA-3’, and DNA extracted from two patients homozygous for rs17315309 wild-type allele T, and for rs17315309 minor allele C, respectively. Cycling conditions were as follows: 94°C, 4 min; 94°C, 30 sec; 61°C, 30 sec; 72°C 30 sec, for 2 cycles, 94°C, 30 sec; 59°C, 30 sec; 72°C 30 sec, for 2 cycles, 94°C, 30 sec; 57°C, 30 sec; 72°C 30 sec, for 38 cycles, 72°C for 10 min. The resulting amplicons were cloned into pGL4.17 vector (Promega, Madison, WI, USA).

NHEKs were co-transfected with the various pGL4.17 vectors and Renilla expression vector and control siRNAs (Life Technologies, Carlsbad, CA) or p53 specific siRNA (Santa Cruz Biotechnology, Santa Cruz, CA) or p63 specific siRNA (Dharmacon, Inc., Lafayette, CO) with lipofectamine2000 and Opti-MEM medium (Life Technologies, Carlsbad, CA). Efficiency of gene knock down was assessed by qRT-PCR (S2 Fig). Cells were grown in KGM medium (Biological Industries, Beit-Haemek, Israel). Twenty-four hours post transfection, cells were harvested and luciferase expression was evaluated using the Dual-Luciferase Reporter Assay System (Promega, Madison, WI, USA) and Tecan Infinite M200 device (Tecan Group Ltd, Männedorf, Switzerland)

### Cytokine secretion measurement

Supernatant collected from NHEKs was evaluated using Elisa assays specific for IL-1α (Human IL-1 alpha/IL-1F1 DuoSet, R&D systems, Minneapolis, MN, USA), TNF-α (Human TNF-alpha Quantikine HS ELISA, R&D systems, Minneapolis, MN, USA) and IL-6 (Human IL-6 DuoSet, R&D systems, Minneapolis, MN, USA). All ELISA assays were read and quantified using Tecan Infinite M200 device (Tecan Group Ltd, Männedorf, Switzerland). For IL-6, a Human Cytokine Array / Chemokine Array 41-Plex (Eve Technologies Corporation, Calgary, Alberta, Canada) was additionally used using a Millpore MILLIPLEX kit (Merck KGaA, Darmstadt, Germany) read by BioPlex 200 (Bio-Rad, Hercules, CA, USA)

### Dispase-based dissociation assay

NHEKs were grown to confluence in triplicates on 6-well plates and exposed to PV or normal serum in a 1:10 dilution or to PV or control IgG antibodies in a final concentration of 65 μg/ml. After 24h the cells were washed twice with PBS, incubated in 2 ml of dispase II (2.4 units/ml, Roche Diagnostics, Basel, Switzerland) at 37°C for 40 minutes and detached from the plate as monolayers. Cell sheets were carefully transferred to 15 ml tube containing 5 ml PBS and subjected to mechanical stress using 5 inversions. The number of fragments was counted by two independent evaluators.

### Statistics

All pairwise comparisons were performed using the 2-tailed Student’s t test, unless otherwise indicated. Differences were considered significant if the P value was less than 0.05.

## Supporting Information

S1 Table rs17315309 genotyping. 1 Risk allele G. 2 Patients of Jewish extraction. 3 Chi-squared test.
(DOCX)

S1 Fig Linkage disequilibrium plot at the ST18 locus. Linkage disequilibrium (LD) values were generated using the Haploview software. LD levels between the various single-nucleotide polymorphisms across this region are represented by variations in the color of the squares, increasing from white (minimal LD) to bright red (maximal LD). Using deep sequencing of the ST18 locus in 16 PV patients as well as additional genotyping of selected genetic variants in 185 PV patients, we identified a haplotype block found to reside within an intron of the ST18 gene and to harbor rs17315309 (arrows). The structure and location of the ST18 gene is indicated.
(TIF)

S2 Fig Efficiency of TP53 or TP63 knock down and ST18 overexpression. (a,b) TP53, TP63 expression in keratinocytes transfected with either TP53 siRNA (si-p53), TP63 siRNA (si-p63) or control siRNA (si-Cont) was measured using qRT-PCR. (c) ST18 expression in keratinocytes transfected with either ST18 expression vector (ST18) or empty control vector (vector) was measured using qRT-PCR. Results (arbitrary units, a.u.) were normalized to GAPDH RNA levels and are expressed as percentage of expression relative to gene expression in control cells ± standard error and represent the mean of two independent experiments (***p<0.005 by 2-tailed t test).
(TIF)

S3 Fig CNBD2 does not promote PV serum-induced cell-cell disadhesion. NHEKs were transfected with a ST18 expression vector (ST18), with a control vector (EV) or with a CNBD2 expression vector (CNBD2) and were grown to confluency in the presence of PV serum or control serum. Epidermal sheets were released from the tissue plates and subjected to mechanical stress as described in Materials and Methods and the resulting fragments were counted. Results are expressed as number of fragments ± SE (*p<0.05, **p<0.01, ***p<0.001 by 2-tailed t test. n.s = not significant)
(TIF)

S4 Fig Correlation between anti-Dsg3 ELISA status of and ST18 genotype. EUROIMMUN anti-Desmoglein 3 ELISA (IgG) test kit was used to determine Dsg3 antibody titers in PV patient serum (n = 43). A cut-off of 20 RU/ml was used to demarcate patients with high or low Dsg3 reactivity. No correlation was found between Dsg3 reactivity and rs17315309 genotype (Chi-square, p value = 0.686).
(TIF)
