# Supplementary material for: Identification of a Functional Risk Variant for Pemphigus Vulgaris in the ST18 Gene
Source: PLoS Genet. 2016 May 5;12(5):e1006008. doi: 10.1371/journal.pgen.1006008 (PMC4858139; doi:10.1371/journal.pgen.1006008)
Supplement: S1 Table — 1 Risk allele G. 2 Patients of Jewish extraction. 3 Chi-squared test. (DOCX) [file pgen.1006008.s001.docx]

**S1 Table**

**rs17315309 genotyping**

| **Genotype^1^** | **Controls^2^** | **Patients^2^** | **P-value^3^** | **Odds ratio** |
| --- | --- | --- | --- | --- |
| AA | 137/183 (74.9%) | 110/185 (59.5%) | 0.000228398 | 2.0314 |
| AG | 42/183 (23.0%) | 60/185 (32.4%) |  |  |
| GG | 4/183 (2.2%) | 15/185 (8.1%) |  |  |

1 Risk allele G

2 Patients of Jewish extraction

3 Chi-squared test.
