# Supplementary figures and images for: Identification of a Functional Risk Variant for Pemphigus Vulgaris in the ST18 Gene
Source: PLoS Genet. 2016 May 5;12(5):e1006008. doi: 10.1371/journal.pgen.1006008 (PMC4858139; doi:10.1371/journal.pgen.1006008)

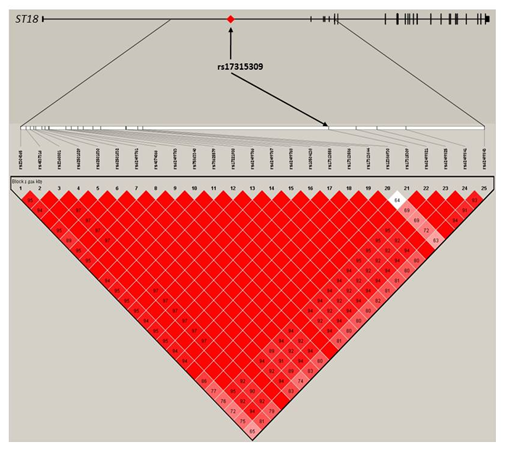

Supplement: S1 Fig — Linkage disequilibrium (LD) values were generated using the Haploview software. LD levels between the various single-nucleotide polymorphisms across this region are represented by variations in the color of the squares, increasing from white (minimal LD) to bright red (maximal LD). Using deep sequencing of the ST18 locus in 16 PV patients as well as additional genotyping of selected genetic variants in 185 PV patients, we identified a haplotype block found to reside within an intron of the ST18 gene and to harbor rs17315309 (arrows). The structure and location of the ST18 gene is indicated. (TIF) [file pgen.1006008.s002.tif]

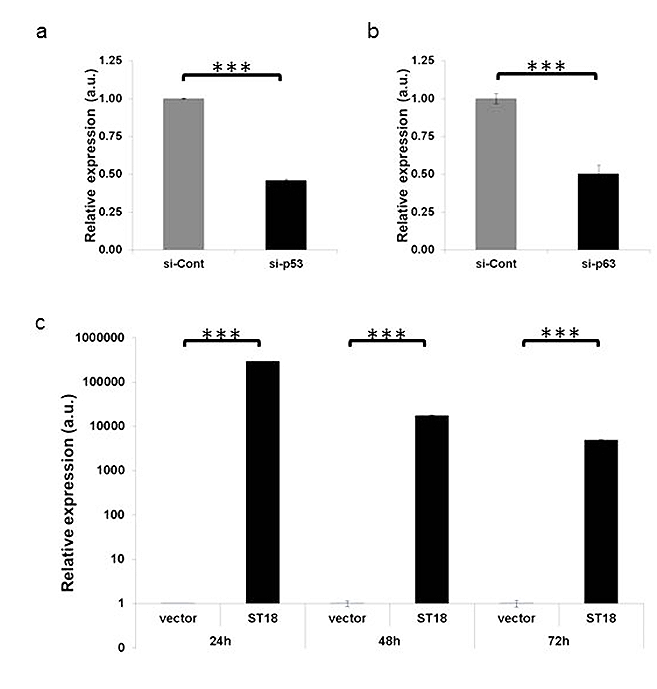

Supplement: S2 Fig — (a,b) TP53, TP63 expression in keratinocytes transfected with either TP53 siRNA (si-p53), TP63 siRNA (si-p63) or control siRNA (si-Cont) was measured using qRT-PCR. (c) ST18 expression in keratinocytes transfected with either ST18 expression vector (ST18) or empty control vector (vector) was measured using qRT-PCR. Results (arbitrary units, a.u.) were normalized to GAPDH RNA levels and are expressed as percentage of expression relative to gene expression in control cells ± standard error and represent the mean of two independent experiments (***p<0.005 by 2-tailed t test). (TIF) [file pgen.1006008.s003.tif]

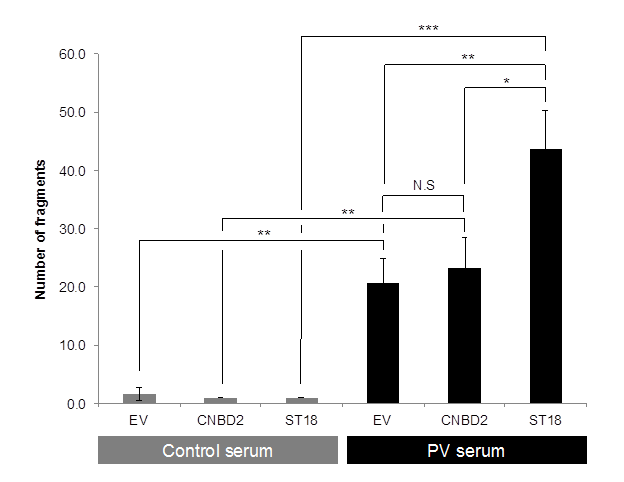

Supplement: S3 Fig — NHEKs were transfected with a ST18 expression vector (ST18), with a control vector (EV) or with a CNBD2 expression vector (CNBD2) and were grown to confluency in the presence of PV serum or control serum. Epidermal sheets were released from the tissue plates and subjected to mechanical stress as described in Materials and Methods and the resulting fragments were counted. Results are expressed as number of fragments ± SE (*p<0.05, **p<0.01, ***p<0.001 by 2-tailed t test. n.s = not significant) (TIF) [file pgen.1006008.s004.tif]

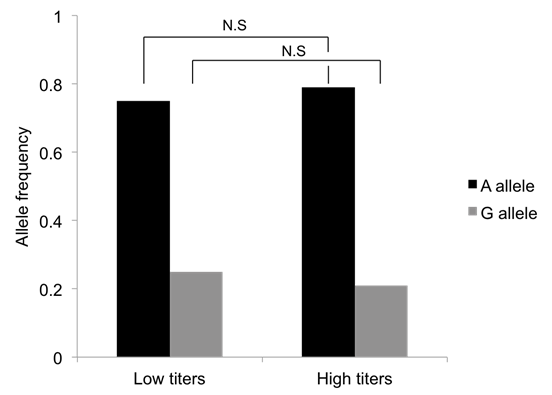

Supplement: S4 Fig — EUROIMMUN anti-Desmoglein 3 ELISA (IgG) test kit was used to determine Dsg3 antibody titers in PV patient serum (n = 43). A cut-off of 20 RU/ml was used to demarcate patients with high or low Dsg3 reactivity. No correlation was found between Dsg3 reactivity and rs17315309 genotype (Chi-square, p value = 0.686). (TIF) [file pgen.1006008.s005.tif]
